# Supplementary material for: Process of use of evidence products by frontline maternal, newborn and child health staff at the facility level in Ghana
Source: PLoS One. 2025 Feb 26;20(2):e0292505. doi: 10.1371/journal.pone.0292505 (PMC11864507; doi:10.1371/journal.pone.0292505)
Supplement: S1 File — (PDF) [file pone.0292505.s001.pdf]

Qualitative Study Tool

INDEPTH INTERVIEW (IDI) GUIDE FOR HEALTH PERSONNEL DIRECTLY INVOLVED IN MATERNAL, NEWBORN AND CHILD HEALTH SERVICES

Topic:

Understanding Knowledge Transfer and Evidence-based Decision Making in Maternal, New born and Child Health Services at the Health Facility Level in Ghana.

Date_________________ Facility type _______________

Facility Ownership________________ Community_________________

Type of Location_________________ Sex of Participant ________________

Occupation____________________ Position of Participant ________________

Time: from_________________ to_________________

Facilitator ____________________________________

In-depth Interview Questions

Opening question

1. What services do you offer?
2. What are the existing frameworks for health service delivery in this facility? (probe for adopted/adapted/ or developed one different from the GHS)
3. What are the existing frameworks/practical guidelines for caring for the clients? (probe for ANC, PNC, Delivery, CWC, Adolescent corner, Immunization, etc)
4. Is your facility's framework for seeking care different from other levels of seeking care?

Use of Evidence-based in Decision Making in MNCH

5. What do you know about the use of guidelines/protocols/policies in health service delivery? (probe for MNCH/RCH)? Why are they important?
6. What are the various guidelines/protocols/policies/research findings you use in providing MNCH/RCH health services for your clients? (Probe for the different clients)? Why?

Understanding the process of use of evidence in clinical practice

1. **Problem Identification**
   i. How do you or your facility identify practice gaps in the provision of MNCH/RCH services?
   ii. [hint:compare your outfit's current practice in MNCH/RCH service provision with existing knowledge].
   iii. How these identified practice gaps addressed?

2. **Adapting Knowledge to Local Context-**
   i. How does your institution adapt existing clinical practice guidelines/protocols/evidence to fit local circumstances?
   ii. [Probe: how it is organized and appropriate to a specific circumstance in MNCH/RCH service provision].

3. **Barriers to Knowledge/Evidence Use**
    i. What factors would you say are constraints in the uptake of knowledge or evidence in your organisation's practice in MNCH/RCH service provision?
    ii. [Probe: knowledge, attitudes, organisational structure and processes, organisational culture, organisational change, resources availability, etc]

4. **Facilitators/Promoters Knowledge/Evidence Use**
    i. What factors would you say are the facilitators/promoters in the uptake of knowledge or evidence in your organisation's practice in MNCH/RCH service provision?
    ii. [Probe: knowledge, attitudes, organisational structure and processes, organisational culture, organisational change, resources availability, etc]

5. **Selecting, Tailoring and Implementing Interventions**
    i. Could you explain how an intervention (based on evidence) is selected for use in your facility? [Probe: What is considered in the selection process and why?, any laid down or standard procedures or SOP, etc]
    ii. How is the process of planning and implementation of an intervention done to bring about the anticipated change in MNCH/RCH service provision?
    iii. [Probe: What are considered the focus at the planning and implementation processes (educational, professional organisational, patient-centred, etc) and why?, etc].

6. **Monitoring of Knowledge Use**
    i. How does your outfit know that whatever intervention(s) is put in place in MNCH/RCH service provision has yielded results?
    ii. [Probe: extent of change, how it is determined, etc].
    iii. What factors may account/accounted for the change (positive or negative) in results?

7. **Sustain Knowledge Use**
    i. How is the outcome or result of an MNCH/RCH intervention further utilised?
    ii. What steps are taken to ensure the positive outcome of the intervention is scaled up?
    iii. What factors aided or will aid in the further utilisation of the knowledge produced in your department/facility?

8. **Other evidence-based related issues**
    i. Whom/which person/institution do you collaborate with in doing research?
    ii. Does your facility have a research unit? [Probe for other places in charge of knowledge transfer or research in facility]
    iii. Could you describe your extent of collaboration with the GHS research centre in your region/municipality/district? [probe for MNCH/RCH research works, probe for other facility, subdistrict, district, municipality, regional, etc]

Closing

9. Is there anything that you feel was not covered in this interview that you'd like to address?

10. Do you have any questions for me?

Thank study participant again for his/her participation in the interview and discuss how his/her answers are important and will be useful in understanding the use of MNCH/RCH guidelines/protocols/policies in service provision in health facilities in Ghana.
